# Superstatistics Applied to Cucurbitaceae DNA Sequences

**DOI:** 10.3390/e26100819

**Published:** 2024-09-25

**Authors:** M. O. Costa, R. Silva, M. M. F. de Lima, D. H. A. L. Anselmo

**Affiliations:** 1 Departamento de Física, Universidade Federal do Rio Grande do Norte, Natal 59072-970, Brazil; marconeoliveiraa@gmail.com (M.O.C.); raimundosilva@fisica.ufrn.br (R.S.); doryh@fisica.ufrn.br (D.H.A.L.A.); 2 Departamento de Física, Universidade do Estado do Rio Grande do Norte, Mossoró 59610-210, Brazil; 3 Departamento de Ciências Vegetais, Universidade Federal Rural do Semi-Árido, Mossoró 59625-900, Brazil

**Keywords:** DNA, *Cucurbitaceae*, non-additive statistics

## Abstract

The short and long statistical correlations are essential in the genomic sequence. Such correlations are long-range for introns, whereas, for exons, these are short. In this study, we employed superstatistics to investigate correlations and fluctuations in the distribution of nucleotide sequence lengths of the *Cucurbitaceae* family. We established a time series for exon sizes to probe these correlations and fluctuations. We used data from the National Center for Biotechnology Information (NCBI) gene database to extract the temporal evolution of exon sizes, measured in terms of the number of base pairs (bp). To assess the model’s viability, we utilized a timescale extraction method to determine the statistical properties of our time series, including the local distribution and fluctuations, which provide the exon size distributions based on the *q*-Gamma and inverse *q*-Gamma distributions. From the Bayesian statistics standpoint, both distributions are excellent for capturing the correlations and fluctuations from the data.

## 1. Introduction

The dynamics associated with complex systems generally present a superposition of many dynamics on different time scales. From a statistical point of view, an approach that naturally captures such a superposition is the so-called superstatistics [1]. Specifically, the main argument for this formalism is to decompose the dynamics of the system into different scales so that its statistical properties follow from a superposition of statistics through a Boltzmann factor $e^{-\beta E}$, where *E* represents the effective energy for each subsystem and a probability density p(β) that results in a fluctuating intensive parameter β. Moreover, one assumes local equilibrium on each scale, achieved at distinct β values, e.g., the dissipation energy and inverse temperature are examples of such parameters [1,2]. Other applications of the superstatistics formalism follow from econophysics [3,4], geophysics [5,6], turbulence [7,8,9], plasmas [10,11,12], and ultra-cold gases [13,14] to high-energy scattering processes [15,16], spin systems [17,18], cosmology [19,20], and stellar systems [21].

In systems of interest in biophysics, there are other connections, such as a superstatistical model (and its corresponding DNA generation algorithm), emulating the rules that dictate the (empirical) nucleotide arrangement properties of some DNA sequences [22]. In Ref. [23], the authors reported an analysis of DNA-binding proteins that exhibit heterogeneous diffusion processes in bacteria [24]. More recently, considering the sequence coding of the human genome, fluctuations in the distribution of the lengths of nucleotide strings have been developed using a stochastic model that provides the distributions of exon size [25].

In the context of the plant genome, on the other hand, there are more than 825 species in the *Cucurbitaceae* family, which is divided into 118 genera. This family contains many plant traits, sexual expression, fruits, and seeds. It has a significant impact as a source of nutrients and fiber [26,27]. The *Cucumis* genus stands out among the well-known genera in this family because it includes two species of commercially significant vegetables that are grown all over the world: cucumber (*Cucumis sativus*) and melon (*Cucumis melo*). As for the *Cucurbita* genus, it has three species that we consider to be the main ones: *Cucurbita maxima*, *Cucurbita moschata*, and *Cucurbita pepo* [28].

The basic number of chromosomes in the *Cucumis* genus can vary from x=7 to x=12; this last value corresponds to the base number of the *Cucurbitaceae* family. There are three types of species: diploid (2x=14 or 24 chromosomes), tetraploid (4x=48 chromosomes), and hexaploid (6x=72 chromosomes) [29,30]. Melon (2x=24 chromosomes) and cucumber (2x=14 chromosomes) are diploid species. The length of the metaphase chromosomes in cucumbers ranges from 1.48μm to 2.31μm [31] and 1.65μm to 3.40μm [32], with the first six chromosomes being metacentric and the smallest submetacentric, according to karyotyping research. As for melon, it is believed that they range from 1.44μm to 2.40μm [33], 1.20 to 2.50μm [34], and from 1.1μm to 1.9μm [35].

The complete sequencing of the cucumber and melon genomes was made possible by developing high-throughput sequencing technology. The genome of a cucumber is estimated to be 367 Mbp [36], but the genome of a melon is estimated to be 450–454 Mbp. The estimated gene count for cucumber is 26,682, with an average gene size of 1046 bp and an average exon count of 4.39. Melon has an estimated 27,427 genes, with an average size of 2776 bp and 5.85 exons per gene [37]. On the other hand, every *Cucurbita* species is a diploid, with 20 chromosomal pairs (2x=40). The plant species of the tribe *Cucurbiteae*, which include *C. pepo*, *C. moschata*, and *C. maxima*, have undergone genome-doubling events [38,39,40]. There are few estimates of the size of the *Cucurbita* genome. However, studies have shown a relatively small genome size. According to estimates, the genomic sizes of *C. maxima*, *C. moschata*, and *C. pepo* are 263.0 Mb, 271.40 Mb, and 269.90 Mb, respectively. The number of genes is 32,076, 32,205, and 27,868 for *C. maxima*, *C. moschata*, and *C. pepo*, respectively [38,39].

The main goal of this paper is to establish a connection between the formalism of superstatistics and the encoding of plant genome sequences. Using this approach, we investigated short-range correlations (SRCs) and fluctuations in the distribution of nucleotide chain lengths based on the temporal scale extraction method, which provides a detailed view of the statistical properties of the time series associated with exon sizes. From this analysis, we observed that local distributions and the local mean fluctuations ξ are well described by *q*-Gamma and inverse *q*-Gamma distributions, supporting the validity of the superstatistics concept in this context. Additionally, to determine which of these distributions offers the best description of the statistical behavior of the time series, we applied Bayesian analysis, which is known for its robustness in various fields. This method has been widely used in areas such as Cosmology [41,42,43,44,45,46], Biophysics [25,47,48,49], and Ecology [50], highlighting its effectiveness for model comparison.

## 2. Materials and Methods

The central premise of the superstatistical approach lies in describing a non-equilibrium heterogeneous system composed of subsystems subject to fluctuations in an intensive parameter. Each subsystem rapidly reaches local equilibrium, characterized by a relaxation time of $\gamma^{-1}$, and maintains this state for a time interval *T* before transitioning to a new value. The superstatistical description can be constructed through a stochastic differential equation of the Langevin type [1], given by

(1) $$\frac{dx}{dt}=-\gamma F\left(x\right)+\zeta L\left(t\right),$$

where the (white Gaussian) noise is denoted by L(t), the friction constant is represented by γ>0, the noise intensity is given by ζ, and the drift force is represented by F(x)=−dV(x)/dx, where V(x) is the potential. Parameters γ and ζ can fluctuate in this theoretical framework so that $\beta =\gamma /\zeta^{2}$ has a probability density function (PDF) given by p(β).

Based on this, we obtain the conditional probability f(x|β) as:

(2) $$f\left(x|\beta \right)=\frac{1}{Z(\beta )}\exp\left[-\beta V(x)\right].$$

where Z(β) is a normalization constant for $\exp\left[-\beta V(x)\right]$ for a given β. The marginal probability p(x) is defined by:

(3) p(x)=∫f(x|β)p(β)dβ.

**Figure 1:**
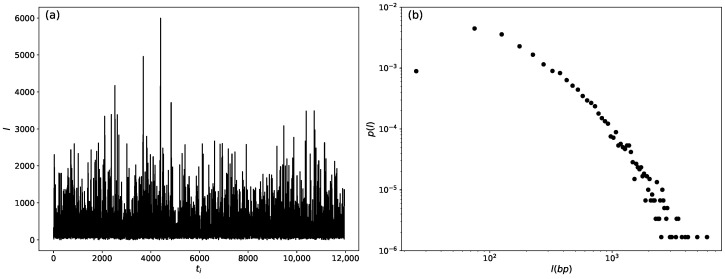
Time series representation and statistical evaluation method. (**a**) Time series for chromosome 01 was created using data from the National Center for Biotechnology Information (NCBI) [51]. The “spatial” series (*l*) represents the spatial displacement along the DNA sequence at “time” *t*. The coordinate position ($t_{i}$) is also linked to a “temporal” index, with i=1,…,n. (**b**) The probability density derived of the “time” series of chromosome 01 in the *C. melo* species.

In our analysis, to test the superstatistical formalism, it is necessary to extract two-time scales, $\gamma^{-1}$ and *T*. Initially, we constructed a set of time series using the complete genomic data of five species from the *Cucurbitaceae* family: *C. melo*, *C. sativus*, *C. maxima*, *C. moschata*, and *C. pepo*, corresponding to the exon lengths along the DNA sequence:

(4) l=l(t),

where the *time series* is defined as random variables $l_{1},\ldots ,l_{i},\ldots ,l_{n}$ indexed in discrete time $t_{1},\ldots ,t_{i},\ldots ,t_{n}$, with the subscript i=1,…,n. Thus, the first value $t_{1}$ can be interpreted as the position of the first exon of length $l_{1}$ on the DNA strand. Similarly, $t_{2}$ represents the position of the second exon of length $l_{2}$. Finally, the *n*-th exon of length $l_{n}$ will have the position $t_{n}$. We utilized the publicly available database provided by the National Center for Biotechnology Information (NCBI) [51]. The time series for chromosome 01 of the *C. melo* species is illustrated in Figure 1a.

To determine statistical information for the time series, we constructed the probability density p(l), as depicted in Figure 1b, using a linear box scale. In this method, the bin sizes for the probability density are incremented using the relation $b_{i}=25\times i$, where $b_{i}$ represents the size of the *i*-th bin for the probability density, with i=1,2,3…,n. This approach allowed us to smooth the fluctuations in the size distributions of exons while preserving the overall shape of the distribution curve.

To ascertain the time scales $\gamma^{-1}$ and *T*, we adopted an approach that analyzes the exponential decay of the autocorrelation function of a time series [4]. In this method, the autocorrelation function is modeled as a combination of two exponential decay functions, as illustrated in the following equation:

(5) $$C\left(\tau \right)=a\exp\left(-t_{1}\tau \right)+b\exp\left(-\tau /t_{2}\right).$$

**Figure 2:**
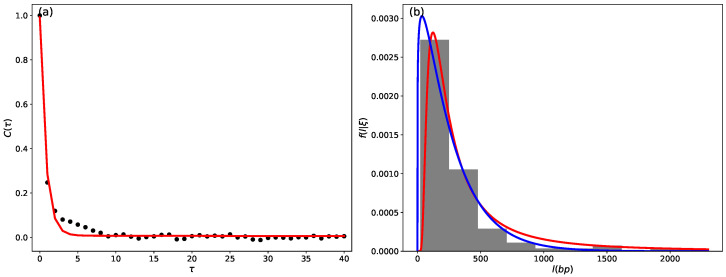
Autocorrelation analysis. (**a**) In the graphical representation, the black points correspond to the autocorrelation of the time series of chromosome 01 in the *C. melo* species. The red line indicates a double exponential fit as defined by Equation (5), with characteristic times $t_{1}=\gamma^{-1}\approx 1.27$ and $t_{2}=T\approx 121$; (**b**) histogram for the local distribution within a window of size *T*. The blue and red curves correspond to the gamma distribution with fitting parameters *k* = 0.3785 and ξ = 768.3620 and the inverse gamma distribution with parameters α = 1.1572 and ξ = 262.2571, respectively.

Here, parameter *a* indicates the maximum amplitude of the autocorrelation function, and parameter *b* is related to the initial decay rate of the autocorrelation function, with $t_{1}$ related to the time scale $\gamma^{-1}$, $t_{2}$ related to the time scale *T*, and τ is lag time. Therefore, the time scales $\gamma^{-1}$ are compared to the time scales *T* through the ratio $t_{2}/t_{1}$. The autocorrelation function plot for chromosome 01 of the *C. melo* species is presented in Figure 2a. In this case, the obtained time scales are $t_{1}=\gamma^{-1}\approx 1.27$ and $t_{2}=T\approx 121$. This demonstrates the existence of two time scales based on the ratio $t_{2}/t_{1}\approx 95$.

When analyzing the time series presented in Figure 1a and windows of size *T*, we observed that the local distribution can be modeled by two distinct distributions: the gamma distribution and the inverse gamma distribution, as depicted in Figure 2b. The following probability density function characterizes the gamma distribution:

(6) $$f_{G}\left(l\right)=\frac{1}{\Gamma (k)}{\left(\frac{k}{\xi }\right)}^{k}x^{k-1}\exp\left(-\frac{k}{\xi }l\right),$$

and the inverse gamma distribution is described by the following equation:

(7) $$f_{IG}\left(l\right)=\frac{\alpha^{\alpha +1}}{\xi \Gamma (\alpha +1)}{\left(\frac{l}{\xi }\right)}^{-\alpha -2}\exp\left(-\frac{\alpha \xi }{l}\right),$$

where *k*, ξ, and α are adjustment parameters, all of which are positive real values.

Notably, the other chromosomes of the examined species had patterns similar to those reported in chromosome 01 of the *C. melo* species. To obtain more details about the temporal durations of these chromosomes, kindly see the provided Table A1 and Table A2 (please see Appendix A).

It is essential to notice that our present approach follows the timescale extraction method and is, therefore, different from the one used in Ref. [25], which is based on the Fokker–Planck and the Langevin equations, to obtain the *q*-Gamma and inverse *q*-Gamma distributions. The choice of the distribution p(ξ) is a crucial concept in superstatistical formalism. To determine this distribution, we followed the method described in the Reference [52] for the local mean ξ. We employed the local variance for gamma and inverse gamma distributions to ascertain the behavior of ξ(t). For the gamma distribution, the local variance is expressed as

(8) $$\xi_{G}\left(t_{0}\right)∼\frac{1}{\sqrt{{\langle l^{2}\rangle }_{t_{0},T}-{\langle l\rangle }_{t_{0},T}^{2}}}.$$

While for the inverse gamma distribution, the local variance is given by

(9) $$\xi_{IG}\left(t_{0}\right)∼\sqrt{{\langle l^{2}\rangle }_{t_{0},T}-{\langle l\rangle }_{t_{0},T}^{2}}.$$

**Figure 3:**
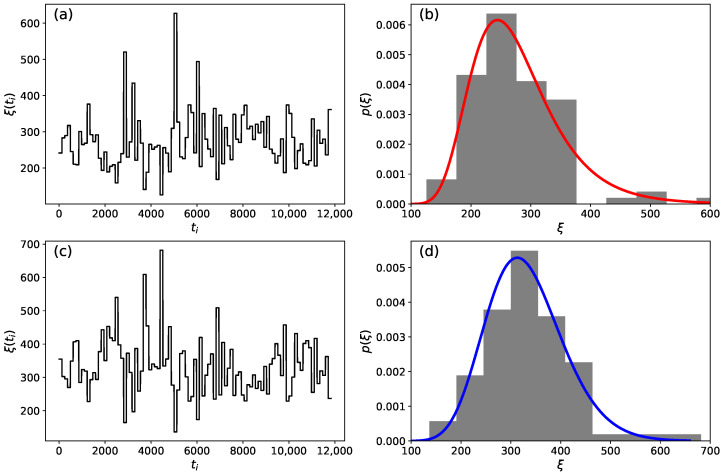
Graphical analysis of the time series ξ(t) for chromosome 01 of the *C. melo* species. (**a**,**c**) depict the time series for ξ(t) obtained from the relationships presented in Equations (8) and (9), respectively. (**b**,**d**) illustrate the histogram describing the distribution for the function p(ξ). In (**b**), the red curve represents the inverse gamma distribution with parameters μ=15.3237 and ω=3989.4215, while in (**d**), the blue curve represents the gamma distribution with parameters δ=18.3461 and ω=18.0439.

Here, ${\langle \cdots \rangle }_{t_{0},\Delta t}=\frac{1}{\Delta t}\int_{t_{0}}^{t_{0}+\Delta t}\cdots dt$ represents integration over an interval of size Δt starting at $t_{0}$. In Figure 3a,c, we depict the behavior of the time series for ξ(t) for chromosome 01 of the *C. melo* species, obtained through the application of Equations (8) and (9). Subsequently, we constructed the probability density histogram for p(ξ), as shown in Figure 3b,d. For this analysis, we compared the histogram of the local variance of the gamma distribution, Figure 3b, with the inverse gamma distribution, which has parameters μ=15.3237 and ω=3989.4215. Regarding the local variance of the inverse gamma distribution, Figure 3d, we compared it with the gamma distribution that has parameters δ=18.3461 and ω=18.0439, defined as:

(10) $$p\left(\xi ;\delta ,\omega \right)=\frac{1}{\omega^{\delta }\Gamma \left(\delta \right)}\xi^{\delta -1}\exp\left(-\xi /\omega \right),$$

and

(11) $$p\left(\xi ;\mu ,\omega \right)=\frac{\omega^{\mu }}{\Gamma (\mu )}\xi^{-\mu -1}\exp\left(-\omega /\xi \right).$$

The fittings of the gamma and inverse gamma distributions provided a meaningful description of the histogram data. These results suggest that both gamma-type and inverse gamma-type statistical distributions are suitable models for describing the data generated by our timescale extraction process. It is important to note that we performed this analysis for all species’ chromosomes.

Then, we proceeded to obtain the *q*-Gamma and inverse *q*-Gamma superstatistics. The mathematical details are given in Appendix B. Here, we limited ourselves to present the expressions. The inverse gamma superstatistics is described by the *q*-Gamma probability density function, which reads:

(12) $$p_{G}\left(l\right)=A_{G}{\left(\frac{l}{\sigma }\right)}^{a}\exp_{q}\left(-\frac{l}{\sigma }\right),$$

and $A_{G}$ is defined as:

(13) $$A_{G}=\frac{{\left(q-1\right)}^{a+1}\Gamma \left(\frac{1}{q-1}\right)}{\sigma \Gamma \left(\frac{1}{q-1}-a-1\right)\Gamma \left(a+1\right)}.$$

Similarly, the gamma superstatistics is described by the inverse *q*-Gamma distribution (we call attention to the reciprocal, inverse nomenclature):

(14) $$p_{IG}\left(l\right)=A_{IG}{\left(\frac{l}{\sigma }\right)}^{-\alpha -2}\exp_{q}\left(-\frac{\sigma }{l}\right).$$

and $A_{IG}$ is given by

(15) $$A_{IG}=\frac{\Gamma \left(\frac{1}{q-1}\right)}{\sigma \Gamma \left(\alpha +1\right)\Gamma \left(\frac{1}{q-1}-\alpha -1\right)}{\left(\frac{1}{q-1}\right)}^{-\alpha -1}.$$

## 3. Results and Discussion

To capture short-range characteristics in the exon size distributions, we used the *q*-Gamma and inverse *q*-Gamma distributions described by Equations (12) and (14) presented in the previous section, fitting them to the data using the Levenberg–Marquardt (LM) algorithm [53]. The results for the *Cucumis* and *Cucurbita* genera are presented in Table A3, Table A4, Table A5, Table A6 and Table A7.

**Figure 4:**
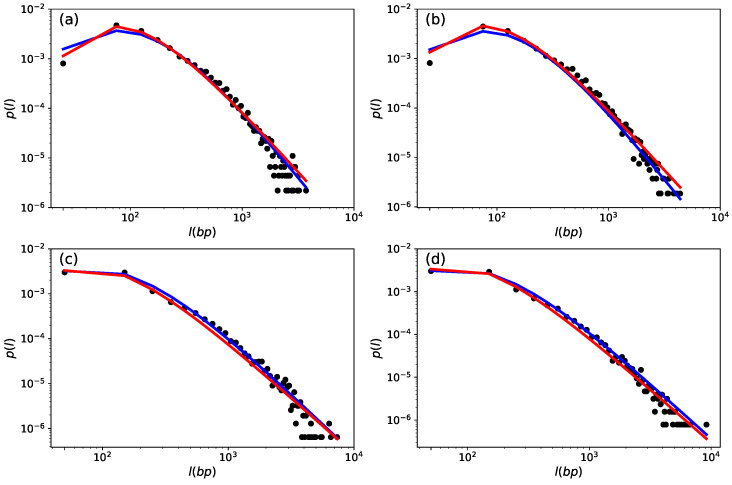
Superstatistical distribution for the string size of chromosomes in the *Cucurbitaceae* family and *Cucumis* genus. The probability distribution of the time series is visually represented by black dots. The red curve illustrates the best fit for the inverse *q*-Gamma distribution. In contrast, the blue line corresponds to the best fit for the *q*-Gamma distribution. (**a**,**b**) show chromosomes 08 and 11, respectively, for the *C. melo* species. (**c**,**d**) show chromosomes 02 and 07, respectively, for the *C. sativus* species.

Figure 4a,b illustrate the distributions for chromosomes 08 and 11 in the *C. melo* species, while Figure 4c,d show the distributions for chromosomes 02 and 07 in the *C. sativus* species. An analysis of Figure 4 reveals that the peak of the distributions in coding regions is around $10^{2}$ base pairs (bp), which is consistent with previous findings in the literature for higher eukaryotes [25]. This pattern is persistent across all chromosomes of the studied species within the *Cucurbitaceae* family. Within the genus *Cucumis*, *C. melo*, Figure 4a,b, presents small discrepancies between the theoretical distributions *q*-Gamma (blue curve) and inverse *q*-Gamma (red curve) with the probability distribution observed from the data (black dots), for values below 50 bp. The same behavior was reported by Costa et al. (2022) [25], in the context of the *Homo sapiens* genome. However, when we evaluated the species *C. sativus*, Figure 4c,d, we did not observe this discrepancy, and the proposed distributions provide a very accurate description of the probability density of the data.

**Figure 5:**
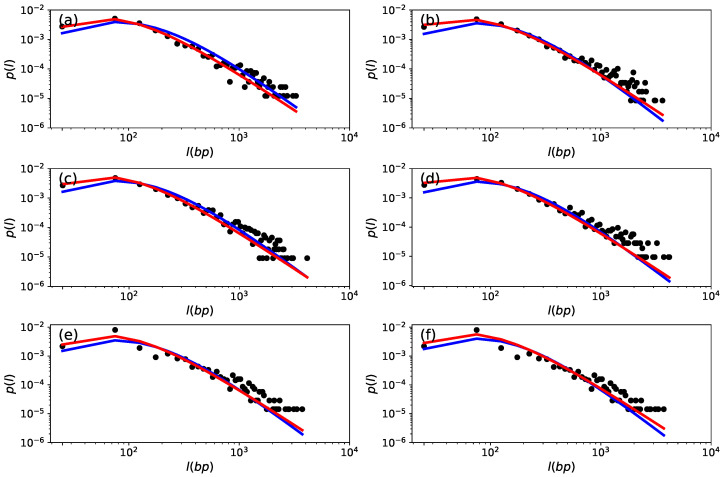
Superstatistical distribution for the string size of the chromosome in the *Cucurbitaceae* family and *Curcubita* genus. The probability distribution of the time series is visually represented by black dots. The red curve illustrates the best fit for the inverse *q*-Gamma distribution. In contrast, the blue line corresponds to the best fit for the *q*-Gamma distribution. (**a**,**b**) show chromosomes 11 and 15, respectively, for the *C. maxima* species. (**c**,**d**) show chromosomes 08 and 15, respectively, for the *C. moschata* species. (**e**,**f**) show chromosomes 04 and 06, respectively, for the *C. pepo* species.

The Figure 5a–f show how the theoretical probability density functions fit the probability density curves observed for some chromosomes of the species *C. maximum*, *C. moschata*, and *C. pepo*, with a similar behavior being observed for the other chromosomes. For these species, we can also observe a slight discrepancy between the behavior of the data (black dots) and the theoretical *q*-Gamma (blue line) and inverse *q*-Gamma (red line) distributions. Here, it is worth stressing that the discrepancies are due to the behavior of the theoretical distributions studied and the observed data. Our intention here is to identify which of the distributions is most suitable for explaining the data, so these discrepancies certainly influence the effectiveness of the evaluated model. However, this does not appear to compromise the quality of the fit to the data.

This work uses Bayesian inference to compare the *q*-Gamma and inverse *q*-Gamma distributions. Bayesian inference is a technique for updating knowledge about the parameters of a model based on new information. This process is related to the dataset *D* and a probabilistic model for a given distribution L(D|θ), known as the likelihood function [54,55], conditioned by the knowledge of the free parameter set θ. Our understanding of θ is quantified by the prior distribution, f(θ). These functions are connected through Bayes’ theorem:

(16) $$P\left(\theta |D\right)=\frac{L(D|\theta )f(\theta )}{\int L(D|\theta )f(\theta )d\theta }.$$

The primary method used in Bayesian inference to decide which model better quantifies the data is by determining the marginal likelihood function. The acquisition of the marginal likelihood function is accomplished by integrating the likelihood function over the parameter space, and it can be expressed as:

(17) ϵ(D)=∫L(D|θ)P(θ)dθ.

Therefore, to perform Bayesian analysis, we will consider that the genomes for the *Cucurbitaceae* family are described by the probability likelihood functions for the *q*-Gamma distribution,

(18) $$L_{G}\left(l\right)=\prod_{i}^{n}p_{G}\left(l_{i}\right)=\prod_{i}^{n}A_{G}{\left(\frac{l_{i}}{\sigma }\right)}^{a}\exp_{q}\left(-\frac{l_{i}}{\sigma }\right),$$

and the inverse *q*-Gamma distribution, given by

(19) $$L_{IG}\left(l\right)=\prod_{i}^{n}p_{IG}\left(l_{i}\right)=\prod_{i}^{n}A_{IG}{\left(\frac{l_{i}}{\sigma }\right)}^{-\alpha -2}\exp_{q}\left(\frac{\sigma }{l_{i}}\right).$$

Thus, when comparing the two models, we used the Bayes factor regarding the ratio of marginal likelihood functions.

(20) $$B_{1,2}=\frac{\epsilon_{1}}{\epsilon_{2}},$$

where $\epsilon_{1}$ is associated with the marginal likelihood function for the *q*-Gamma distribution and $\epsilon_{2}$ is associated with the marginal likelihood function for the inverse *q*-Gamma distribution.

To quantify whether the model presents favorable evidence, we used Jeffreys’ scale of evidence for the Bayes factor [56], presented in Table 1. This table represents an empirically calibrated scale for values of $\ln(B_{1,2})$: $\ln(B_{1,2})>1$ indicates favorable evidence for the inverse *q*-Gamma distribution; $\ln(B_{1,2})<-1$ represents favorable evidence for the *q*-Gamma distribution, and $-1\leq \ln(B_{1,2})\leq 1$ represents inconclusive evidence, making it impossible to determine which model best describes the data set. This work uses the inverse *q*-Gamma distribution as a reference model.

**Figure 6:**
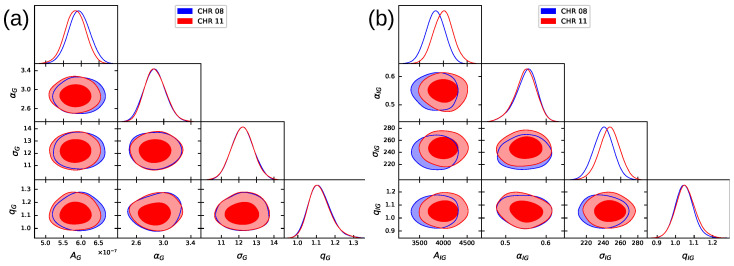
Results of the Bayesian inference process, showing projections of the posterior distributions for the free parameters of the *q*-Gamma and inverse *q*-Gamma distributions for the *Cucumis* genus. (**a**,**b**) present the distributions for chromosomes 08 and 11 of the *C. melo* species, respectively.

**Figure A3:**
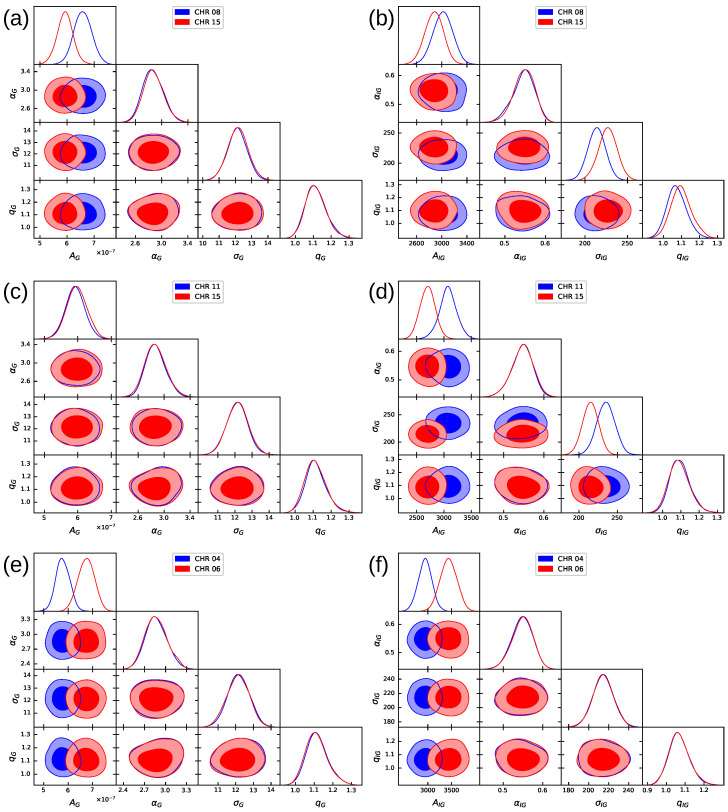
Results of the Bayesian inference process, showing projections of the posterior distributions for the free parameters of the *q*-Gamma and inverse *q*-Gamma distributions for the *Curcubita* genus. (**a**,**b**) present the distributions for chromosomes 11 and 15 of the *C. maxima* species, respectively. (**c**,**d**) show the distributions for chromosomes 08 and 15 of the *C. moschata* species, respectively. (**e**,**f**) show the distributions for chromosomes 04 and 06 of the *C. pepo* species, respectively.

To determine the Bayesian evidence associated with the theoretical probability distributions studied, whether Equation (12) (*q*-Gamma) or Equation (14) (inverse *q*-Gamma), we used the Ultranest [57] package, a Bayesian analysis tool that employs the concept of nested sampling to calculate Bayesian evidence and posterior distributions [58,59,60]. We constructed scatter plots to visualize the relationships between the parameters of the proposed theoretical models. Figure 6 shows the interaction between the parameters of Equation (12) (panel a) and Equation (14) (panel b), for the species *C. melo*. The same behavior can be observed for all species studied (see Figure A3 in Appendix A).

The previous distribution considers the model under study’s configurable parameters. Consequently, this study phase is critical since it will establish the trajectory of the quest for ideal parameters. This factor can directly impact the model’s capacity for prediction. Prior distributions can be chosen in a variety of ways when there is little prior knowledge; for instance, a Uniform distribution is utilized, which specifies the minimum and maximum values for the parameters and indicates that any value falling between these two has an equal chance of being the best parameter to describe the data’s behavior. When dealing with a normal (Gaussian) distribution, the most likely value of the adjustable parameter (Gaussian peak) and the standard deviation of the error estimate are required to determine the algorithm’s maximum search limit. It is important to note that, in this instance, it is inferred that the likelihood of identifying the ideal parameter to describe the observed data decreases with increasing distance from the given average value. One can also utilize other prior distributions; see [61]. In this study, we used normal distributions as priors. The values determined for each parameter of the investigated models are detailed in Table 2. Figure 6 can allow us to visualize the distributions of the parameters in question.

Therefore, the next stage of our research will evaluate how well the models match the observational data. We must apply the Bayes factor to compare the Bayesian evidence of the theoretical models under examination. Taking the ln on both sides of Equation (20), we obtain that $\ln B_{1,2}=\ln\epsilon_{1}-\ln\epsilon_{2}$. Consequently, all that is left to do is compute the difference between the evidence of the models and apply the Table 1 interpretation. The Ultranest package provides each model’s logarithm of the evidence. Table 1 shows the results for all species’ chromosomes *C. melo* and *C. sativus*. All other species showed similar behavior (see Table A8).

For chromosome 1 of the species *C. melo*, we computed the differences between the evidence $\ln\epsilon_{1}$ and $\ln\epsilon_{2}$ (see Table 3). This yielded the value $\ln(B_{1,2})=-0.065\pm 0.022$. We can conclude that both models are equally good at explaining the data’s behavior and that there is no statistically significant difference between them based on Jeffreys’ interpretation of the Bayes factor. A similar behavior was observed for the other chromosomes of the *C. melo* species as well as those of *C. sativus*, *C. maxima*, *C. moschata*, and *C. pepo*. Therefore, our results indicate that the q−gamma distribution and the inverse q−gamma distribution can be used to study the genome length distributions of the species analyzed here.

## 4. Conclusions

In this article, the length distributions of exon sequences of five species of the *Cucurbitaceae* family are analyzed: *C. melo*, *C. sativus*, *C. maxima*, *C. moschata*, and *C. pepo*. To perform this analysis, we implemented the timescale extraction method, which provided the distributions of exon sizes by considering the time series’ statistical properties. Based on the local distribution and fluctuations in the intensive parameter ξ, it was possible to show that the exon size distributions followed the *q*-Gamma and inverse *q*-Gamma distributions. Both distributions were obtained by analyzing data windows proportionate in size to the time scale *T*.

We used each chromosome belonging to the species studied in this research, constructed a time series from the datasets, and performed analyses. Specifically, we examined the exon size distributions, expressed in base pairs (bp), using the *q*-Gamma and inverse *q*-Gamma functions. As reported by Costa et al. [25], these functions also exhibited a range in which they deviated from the data when the values were small, below 50 bp. These distributions were able to capture low-order correlations in all investigated species.

The two probability distributions proposed in this study naturally incorporated the extensive parameter *q*, which, in the statistical generalization proposed by Tsallis, evaluates the autocorrelations present in the system under investigation. When q→1, the conventional statistical pattern is recovered; each part of the system is independent [62]. For the genus *Cucumis*, the studied species exhibited an average $q_{G}$ of 1.1726±0.0064 for the *q*-Gamma distribution and an average $q_{IG}$ of 1.0919±0.0032 for the inverse *q*-Gamma distribution. In the case of the genus *Cucurbita*, the average $q_{G}$ for the *q*-Gamma distribution was 1.1727±0.0071, while the average $q_{IG}$ for the inverse *q*-Gamma distribution was 1.1316±0.0216. These results demonstrate short-range correlations, as described in the literature for the coding part of the genome of higher eukaryotes [25,47,49].

Still in the context of the analysis of plant genome lengths, we can observe that our results point to well-defined behavior for the entropic index *q* for all chromosomes and all species studied, which may indicate a specific standard biological signature among plants, a behavior that has already been observed for plant, animal, and viral genomes, even within other generalized statistical contexts, even when we consider the sequences of exons [47,63], introns [48], or proteins [64,65].

We implemented a Bayesian inference selection method to compare the proposed distributions, *q*-Gamma and inverse *q*-Gamma. In Figure 6 and Figure A3, we present triangle plots constructed with confidence regions for the parameters. Based on the data provided in Table 3 and Table A8, it is evident that the *q*-Gamma and inverse *q*-Gamma distributions share equal statistical weight in modeling the size distribution for the investigated species within the *Cucurbitaceae* family. It is worth noting that the investigated distributions emerge naturally from the proposed model; moreover, when we used other priors, the results differed from those presented here.

## Figures and Tables

**Table 1:** The Jeffreys’ scale for interpreting the Bayes factor. The first column represents the logarithm of the Bayes factor limit values, while the second column is the interpretation of the evidence’s strength over the appropriate threshold.

$\ln B_{1,2}$	Interpretation
Greater than 5	Strong evidence for model 01
(2.5, 5)	Moderate evidence for model 01
(1, 2.5)	Weak evidence for model 01
(−1,1)	Inconclusive
(−2.5,−1)	Weak evidence for model 02
(−5, −2.5)	Moderate evidence for model 02
Less than −5	Strong evidence for model 02

**Table 2:** The table presents the normal priors used for the adjustment parameters in the models described by Equations (12) and (14). The first column indicates the parameter, and the second and third specify the model in which the parameters are part, i.e., q−gamma and inverse q−gamma, respectively.

Parameters	q-Gamma		Inverse q-Gamma	
	**Mean**	**Standard Deviation**	**Mean**	**Standard Deviation**
$A_{G}$	$6.05\times 10^{-7}$	$7\times 10^{-8}$	-	-
$\alpha_{G}$	2.99	0.30	-	-
$\sigma_{G}$	12.29	1.23	-	-
$q_{G}$	1.17	0.12	-	-
$A_{IG}$	-	-	$3.25\times 10^{3}$	$3.25\times 10^{2}$
$\alpha_{IG}$	-	-	0.52	0.05
$\sigma_{IG}$	-	-	222.67	22.30
$q_{IG}$	-	-	1.12	0.13

**Table 3:** The table presents the Bayesian evidence and the Bayes factors for each chromosome of the *C. melo* and *C. sativus* species. The column $\ln(\epsilon_{1})$ presents the Bayesian evidence for the *q*-Gamma distribution, $\ln(\epsilon_{2})$ presents the Bayesian evidence for the inverse *q*-Gamma distribution, and $\ln(B_{1,2})$ presents the Bayes factor.

	*C. melo*			*C. sativus*		
**CHR**	$\ln\left(\epsilon_{1}\right)$	$\ln\left(\epsilon_{2}\right)$	$\ln\left(B_{1,2}\right)$	$\ln\left(\epsilon_{1}\right)$	$\ln\left(\epsilon_{2}\right)$	$\ln\left(B_{1,2}\right)$
1	−0.085±0.019	−0.020±0.010	−0.065±0.009	−0.061±0.040	−0.009±0.009	−0.052±0.030
2	−0.105±0.026	−0.021±0.010	−0.084±0.016	−0.062±0.040	−0.009±0.009	−0.052±0.030
3	−0.077±0.024	−0.020±0.009	−0.056±0.014	−0.052±0.032	−0.009±0.009	−0.042±0.022
4	−0.106±0.036	−0.021±0.009	−0.085±0.026	−0.061±0.046	−0.009±0.009	−0.051±0.036
5	−0.087±0.026	−0.020±0.010	−0.067±0.016	−0.068±0.034	−0.009±0.009	−0.059±0.024
6	−0.095±0.023	−0.020±0.010	−0.074±0.013	−0.069±0.039	−0.009±0.009	−0.059±0.030
7	−0.107±0.029	−0.020±0.010	−0.087±0.019	−0.068±0.035	−0.010±0.009	−0.058±0.025
8	−0.097±0.025	−0.020±0.010	−0.077±0.015	-	-	-
9	−0.091±0.024	−0.020±0.010	−0.071±0.014	-	-	-
10	−0.092±0.028	−0.020±0.010	−0.071±0.019	-	-	-
11	−0.081±0.027	−0.021±0.010	−0.060±0.017	-	-	-
12	−0.077±0.023	−0.020±0.010	−0.057±0.013	-	-	-

**Figure A1:**
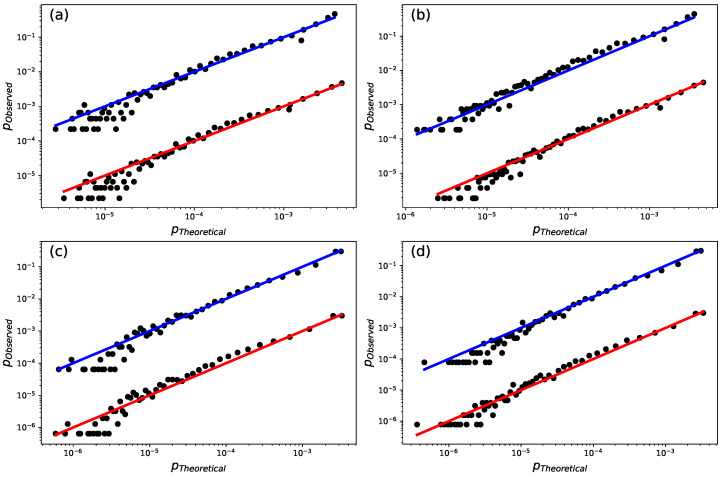
*Quantile–quantile* representation of the superstatistical distributions for nucleotide chain lengths in the chromosomes of the family *Cucurbitaceae*, genus *Cucumis*. The black dots represent the observed data, while the red line illustrates the theoretical distribution based on the inverse *q*-Gamma. In contrast, the blue line represents the theoretical *q*-Gamma distribution. For better visualization, the values of the *q*-Gamma distribution were shifted by a factor of $10^{2}$. (**a**,**b**) show chromosomes 08 and 11, respectively, for the species *C. melo*, while (**c**,**d**) correspond to chromosomes 02 and 07, respectively, for the species *C. sativus*.

**Figure A2:**
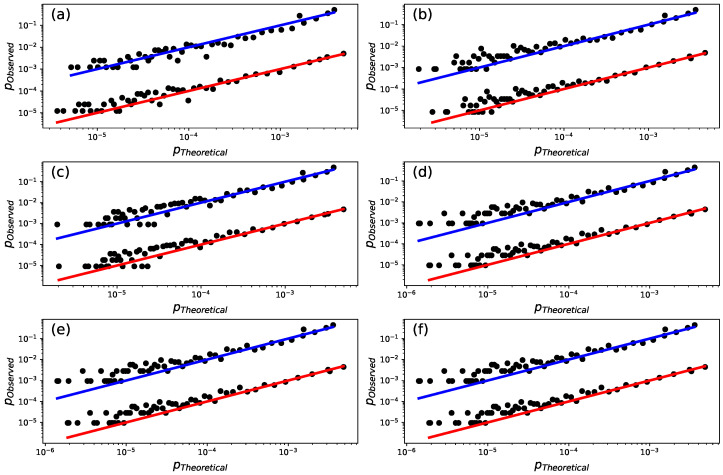
*Quantile–quantile* representation of the superstatistical distributions for nucleotide chain lengths in the chromosomes of the family *Cucurbitaceae*, genus *Cucurbita*. The black dots represent the observed data, while the red line illustrates the theoretical distribution based on the inverse *q*-Gamma. In contrast, the blue line represents the theoretical *q*-Gamma distribution. For better visualization, the values of the *q*-Gamma distribution were shifted by a factor of $10^{2}$. (**a**,**b**) show chromosomes 11 and 15, respectively, for the species *C. maxima*. (**c**,**d**) show chromosomes 08 and 15, respectively, for the species *C. moschata*. (**e**,**f**) show chromosomes 04 and 06, respectively, for the species *C. pepo*.

## Data Availability

The DNA code data that support the results of this study are available at the National Center for Biotechnology Information—NCBI [51] (https://www.ncbi.nlm.nih.gov/, accessed on 22 October 2023).

## Appendix A

In the main text, to determine the time scales $\gamma^{-1}$ and *T*, we analyzed the exponential decay of the autocorrelation function of a time series, modeling it as a combination of two exponential functions (Equation (5)). $t_{1}$ is related to the scale $\gamma^{-1}$ and $t_{2}$ to the scale *T*, with τ being the lag time. In chromosome 01 of *C. melo*, we obtained $t_{1}\approx 1.27$ and $t_{2}\approx 121$, showing two time scales with the ratio $t_{2}/t_{1}\approx 95$. The additional chromosomes of this species and every other species included in the research are detailed in Table A1 and Table A2.

**Table A1:** The autocorrelation analysis described by Equation (5) applied to the *C. melo* and *C. sativus* species.

*C. melo*							*C. sativus*					
**CHR**	a	$t_{1}$	b	$t_{2}$	T	$t_{2}/t_{1}$	a	$t_{1}$	b	$t_{2}$	T	$t_{2}/t_{1}$
1	0.9854	1.27	0.0081	120.50	121	95.28	0.9940	1.72	0.0039	249.70	250	145.35
2	0.9933	1.38	0.0029	183.25	183	132.32	0.9857	1.60	0.0116	160.38	160	100.31
3	0.9839	1.27	0.0106	116.64	117	92.13	0.9941	1.50	0.0027	156.62	157	104.74
4	0.9865	1.25	0.0052	145.34	145	116.28	0.9932	1.64	0.0043	138.75	139	84.60
5	0.9877	1.34	0.0072	179.80	180	133.93	0.9898	1.54	0.0065	123.02	123	79.97
6	0.9868	1.22	0.0054	137.93	138	113.21	0.9908	1.58	0.0068	114.65	115	72.60
7	0.9863	1.16	0.0031	173.42	173	149.27	0.9948	1.64	0.0034	155.60	156	95.30
8	0.9858	1.32	0.0081	122.67	123	93.04	-	-	-	-	-	-
9	0.9905	1.30	0.0030	168.08	168	129.23	-	-	-	-	-	-
10	0.9940	1.22	0.0008	280.17	280	230.45	-	-	-	-	-	-
11	0.9869	1.23	0.0067	154.90	155	126.32	-	-	-	-	-	-
12	0.9886	1.27	0.0040	160.76	161	126.67	-	-	-	-	-	-

**Table A2:** The autocorrelation analysis described by Equation (5) applied to the *C. maxima*, *C. moschata*, and *C. pepo* species.

*C. maxima*							*C. moschata*						*C. pepo*					
**CHR**	a	$t_{1}$	b	$t_{2}$	T	$t_{2}/t_{1}$	a	$t_{1}$	b	$t_{2}$	T	$t_{2}/t_{1}$	a	$t_{1}$	b	$t_{2}$	T	$t_{2}/t_{1}$
1	0.9838	2.10	0.0151	87.18	87	41.45	0.9870	1.68	0.0127	66.08	66	39.29	0.9637	1.79	0.0345	144.16	144	80.27
2	1.0010	1.62	−0.0025	120.33	120	74.17	0.9835	1.62	0.0155	63.86	64	39.51	0.9690	1.50	0.0272	57.78	58	38.67
3	0.9876	1.72	0.0111	68.62	69	40.05	0.9951	1.50	0.0035	81.42	81	53.96	0.8891	1.54	0.1083	74.48	74	48.08
4	0.9975	1.94	0.0021	84.08	84	43.23	0.9891	2.09	0.0108	65.49	65	31.04	0.9620	1.94	0.0365	79.26	79	40.81
5	0.9943	2.03	0.0049	76.55	77	37.88	0.9970	1.82	0.0016	89.11	89	48.98	0.9893	1.79	0.0099	70.03	70	39.17
6	0.9960	1.84	0.0033	82.88	83	45.18	1.0050	1.43	−0.0054	80.81	81	56.68	0.9605	1.25	0.0360	56.63	57	45.56
7	0.9834	1.66	0.0155	66.41	66	39.66	0.9821	1.25	0.0095	71.70	72	57.78	0.9467	1.04	0.0326	57.26	57	55.07
8	0.9891	1.74	0.0097	71.66	72	41.45	0.9994	1.54	−0.0028	120.01	120	77.82	0.9228	0.90	−0.0143	76.19	76	84.44
9	0.9897	2.12	0.0099	70.76	71	33.52	1.0010	1.83	−0.0022	119.90	120	65.43	0.9496	1.97	0.0487	54.24	54	27.44
10	0.9925	1.95	0.0066	73.13	73	37.49	0.9845	1.62	0.0155	65.15	65	40.07	0.9903	1.64	0.0062	85.31	85	51.99
11	0.9945	2.17	0.0043	79.83	80	36.88	0.9927	1.70	0.0036	79.58	80	47.00	0.9424	1.60	0.0545	61.27	61	38.10
12	0.9900	1.29	0.0014	86.92	87	67.44	0.9832	1.60	0.0130	68.19	68	42.39	0.9696	1.78	0.0271	57.90	58	32.60
13	0.9889	1.78	0.0080	71.82	72	40.36	0.9233	0.90	−0.0154	67.00	67	74.44	0.9874	1.62	0.0079	72.82	73	44.95
14	0.9924	1.78	0.0073	72.33	72	40.40	0.9897	1.73	0.0099	68.65	69	39.93	0.9579	1.82	0.0421	54.78	55	30.22
15	0.9946	2.25	0.0062	75.28	75	33.27	0.9835	2.14	0.0157	63.94	64	29.85	0.9917	1.74	0.0061	74.88	75	43.23
16	0.7720	1.99	0.0217	60.62	61	30.61	0.9929	1.98	0.0075	71.98	72	36.38	0.9808	1.52	0.0126	68.38	68	44.65
17	0.9868	2.14	0.0146	63.51	64	29.88	0.9689	1.90	0.0300	120.40	120	63.26	0.9879	1.45	0.0072	120.03	120	82.99
18	0.9853	2.15	0.0143	65.26	65	30.30	0.9802	1.84	0.0202	93.33	93	50.43	0.9422	2.18	0.0571	54.74	55	25.23
19	0.9809	2.07	0.0188	61.13	61	29.44	0.9700	1.63	0.0272	71.94	72	44.20	0.9340	1.58	0.0640	53.82	54	34.13
20	0.9903	2.05	0.0102	70.69	71	34.58	0.9804	1.61	0.0161	65.08	65	40.40	0.9908	1.82	0.0076	75.82	76	41.80

The optimal parameters found for the q−gamma and inverse q−gamma distributions via Equations (A7) and (A16), respectively, are described in Table A3, Table A4, Table A5, Table A6 and Table A7, for all chromosomes of all five species studied. These values can provide us with important information about the behavior of the observational data.

Figure A1 and Figure A2 present the quantile–quantile analysis applied to species belonging to the genera *Cucumis* and *Cucurbita*. Each subplot compares theoretical distributions, represented on the horizontal axis, and empirical distributions, observed in the data, represented on the vertical axis. In the context of the analyses performed, the data exhibit behavior that can be adequately modeled by *q*-Gamma and inverse *q*-Gamma distributions, represented by the blue and red curves, respectively. It highlights that both distributions are capable of reproducing the observed behavior.

Figure A3 shows the interaction between the parameters of Equations (12) and (14). It presents the projections of the posterior distributions for the free parameters of the distributions mentioned above for the genus *Curcubita*. In addition, (a,b) present the distributions for chromosomes 11 and 15 of the species *C. maxima*, respectively; (c,d) show the distributions for chromosomes 08 and 15 of the species *C. moschata*, respectively; and (e,f) show the distributions for chromosomes 04 and 06 of the species *C. pepo*, respectively. All other chromosomes present the same behavior.

**Table A3:** The table shows the best values of the parameters $A_{G}$, $a_{G}$, $\sigma_{G}$, and $q_{G}$ for the *q*-Gamma distribution mentioned in Equation (12), as well as the values of $A_{IG}$, $\alpha_{IG}$, $\sigma_{IG}$, and $q_{IG}$ for the inverse *q*-Gamma distribution described in Equation (14), applied to the *C. melo* species.

	*q*-Gamma				Inverse *q*-Gamma			
**CHR**	$A_{G}$	$a_{G}$	$\sigma_{G}$	$q_{G}$	$A_{\mathrm{IG}}$	$\alpha_{\mathrm{IG}}$	$\sigma_{\mathrm{IG}}$	$q_{\mathrm{IG}}$
1	$5.865\times 10^{-7}\pm 2.881\times 10^{-8}$	2.982±0.142	12.318±0.620	1.172±0.053	$4.135\times 10^{3}\pm 2.056\times 10^{2}$	0.528±0.026	243.701±12.038	1.090±0.057
2	$5.809\times 10^{-7}\pm 2.868\times 10^{-8}$	2.986±0.149	12.344±0.590	1.177±0.053	$4.526\times 10^{3}\pm 2.242\times 10^{2}$	0.524±0.026	257.184±12.575	1.091±0.052
3	$5.928\times 10^{-7}\pm 2.861\times 10^{-8}$	2.975±0.151	12.329±0.608	1.169±0.051	$3.931\times 10^{3}\pm 1.947\times 10^{2}$	0.525±0.026	235.742±11.563	1.097±0.053
4	$5.806\times 10^{-7}\pm 2.896\times 10^{-8}$	2.983±0.145	12.352±0.605	1.170±0.056	$4.389\times 10^{3}\pm 2.166\times 10^{2}$	0.525±0.026	250.523±12.558	1.091±0.054
5	$5.985\times 10^{-7}\pm 3.022\times 10^{-8}$	2.984±0.151	12.365±0.580	1.165±0.055	$3.849\times 10^{3}\pm 1.979\times 10^{2}$	0.525±0.026	232.731±11.460	1.092±0.054
6	$5.929\times 10^{-7}\pm 2.992\times 10^{-8}$	2.990±0.149	12.296±0.625	1.169±0.053	$3.819\times 10^{3}\pm 1.863\times 10^{2}$	0.524±0.024	232.298±11.564	1.092±0.053
7	$5.922\times 10^{-7}\pm 2.853\times 10^{-8}$	2.986±0.140	12.361±0.622	1.172±0.055	$3.828\times 10^{3}\pm 1.903\times 10^{2}$	0.527±0.025	232.823±11.860	1.096±0.057
8	$5.959\times 10^{-7}\pm 2.865\times 10^{-8}$	2.986±0.143	12.319±0.603	1.171±0.054	$3.899\times 10^{3}\pm 1.999\times 10^{2}$	0.526±0.027	233.974±11.753	1.083±0.053
9	$5.868\times 10^{-7}\pm 2.902\times 10^{-8}$	2.989±0.146	12.337±0.626	1.171±0.054	$3.993\times 10^{3}\pm 1.949\times 10^{2}$	0.526±0.027	239.356±12.004	1.088±0.054
10	$5.906\times 10^{-7}\pm 2.817\times 10^{-8}$	2.979±0.139	12.320±0.614	1.170±0.055	$3.739\times 10^{3}\pm 1.844\times 10^{2}$	0.524±0.027	229.712±11.908	1.094±0.054
11	$5.843\times 10^{-7}\pm 2.860\times 10^{-8}$	2.983±0.149	12.341±0.631	1.170±0.056	$4.068\times 10^{3}\pm 1.984\times 10^{2}$	0.524±0.026	241.484±12.251	1.098±0.055
12	$5.836\times 10^{-7}\pm 2.805\times 10^{-8}$	2.970±0.139	12.325±0.613	1.170±0.055	$4.145\times 10^{3}\pm 2.126\times 10^{2}$	0.527±0.026	244.823±12.665	1.093±0.054

**Table A4:** The same procedures as in Table A3 were applied to the *C. sativus* species.

	*q*-Gamma				Inverse *q*-Gamma			
**CHR**	$A_{G}$	$a_{G}$	$\sigma_{G}$	$q_{G}$	$A_{\mathrm{IG}}$	$\alpha_{\mathrm{IG}}$	$\sigma_{\mathrm{IG}}$	$q_{\mathrm{IG}}$
1	$5.719\times 10^{-7}\pm 2.955\times 10^{-8}$	2.990±0.146	12.282±0.563	1.175±0.054	$3.255\times 10^{3}\pm 1.651\times 10^{2}$	0.527±0.026	234.521±11.537	1.090±0.054
2	$5.714\times 10^{-7}\pm 2.835\times 10^{-8}$	2.990±0.142	12.286±0.596	1.176±0.054	$3.554\times 10^{3}\pm 1.777\times 10^{2}$	0.525±0.025	241.540±12.020	1.089±0.053
3	$5.696\times 10^{-7}\pm 2.869\times 10^{-8}$	2.986±0.143	12.307±0.612	1.176±0.054	$3.583\times 10^{3}\pm 1.861\times 10^{2}$	0.524±0.025	244.520±12.068	1.090±0.055
4	$5.735\times 10^{-7}\pm 2.794\times 10^{-8}$	2.989±0.146	12.277±0.637	1.168±0.053	$3.252\times 10^{3}\pm 1.650\times 10^{2}$	0.525±0.026	233.304±11.927	1.092±0.054
5	$5.724\times 10^{-7}\pm 2.756\times 10^{-8}$	3.003±0.146	12.277±0.592	1.169±0.054	$3.551\times 10^{3}\pm 1.807\times 10^{2}$	0.524±0.026	243.650±12.022	1.091±0.053
6	$5.716\times 10^{-7}\pm 2.868\times 10^{-8}$	2.973±0.151	12.290±0.615	1.172±0.056	$3.269\times 10^{3}\pm 1.652\times 10^{2}$	0.524±0.025	233.519±11.283	1.091±0.051
7	$5.696\times 10^{-7}\pm 2.894\times 10^{-8}$	2.983±0.144	12.262±0.617	1.179±0.056	$3.693\times 10^{3}\pm 1.813\times 10^{2}$	0.522±0.026	245.726±11.915	1.092±0.055

**Table A5:** The same procedures as in Table A3 were applied to the *C. maxima* species.

	*q*-Gamma				Inverse *q*-Gamma			
**CHR**	$A_{G}$	$a_{G}$	$\sigma_{G}$	$q_{G}$	$A_{\mathrm{IG}}$	$\alpha_{\mathrm{IG}}$	$\sigma_{\mathrm{IG}}$	$q_{\mathrm{IG}}$
1	$1.456\times 10^{-6}\pm 7.438\times 10^{-8}$	2.749±0.144	13.207±0.706	1.175±0.055	$3.361\times 10^{3}\pm 1.596\times 10^{2}$	0.527±0.026	237.645±11.970	1.158±0.056
2	$6.285\times 10^{-7}\pm 3.048\times 10^{-8}$	2.984±0.147	12.270±0.621	1.169±0.053	$3.596\times 10^{3}\pm 1.768\times 10^{2}$	0.524±0.026	254.482±12.562	1.164±0.058
3	$6.220\times 10^{-7}\pm 3.159\times 10^{-8}$	2.985±0.144	12.289±0.630	1.174±0.053	$3.001\times 10^{3}\pm 1.612\times 10^{2}$	0.523±0.026	216.471±10.948	1.137±0.054
4	$6.231\times 10^{-7}\pm 3.137\times 10^{-8}$	2.983±0.143	12.277±0.614	1.174±0.055	$3.047\times 10^{3}\pm 1.387\times 10^{2}$	0.528±0.025	215.570±10.733	1.134±0.056
5	$1.468\times 10^{-6}\pm 7.270\times 10^{-8}$	2.606±0.133	18.068±0.924	1.172±0.057	$3.569\times 10^{3}\pm 1.833\times 10^{2}$	0.525±0.028	241.221±12.369	1.148±0.058
6	$6.415\times 10^{-7}\pm 3.254\times 10^{-8}$	2.974±0.153	12.294±0.614	1.173±0.055	$3.014\times 10^{3}\pm 1.514\times 10^{2}$	0.526±0.026	208.958±10.860	1.121±0.055
7	$6.243\times 10^{-7}\pm 3.146\times 10^{-8}$	2.976±0.146	12.302±0.606	1.176±0.055	$2.959\times 10^{3}\pm 1.476\times 10^{2}$	0.527±0.026	212.403±11.015	1.124±0.056
8	$6.593\times 10^{-7}\pm 3.283\times 10^{-8}$	2.988±0.143	12.231±0.601	1.170±0.055	$3.071\times 10^{3}\pm 1.503\times 10^{2}$	0.522±0.026	208.611±10.280	1.123±0.055
9	$6.020\times 10^{-7}\pm 3.110\times 10^{-8}$	2.983±0.148	12.290±0.607	1.171±0.053	$3.607\times 10^{3}\pm 1.795\times 10^{2}$	0.525±0.025	254.313±12.365	1.163±0.058
10	$1.462\times 10^{-6}\pm 7.095\times 10^{-8}$	2.696±0.134	12.306±0.622	1.208±0.055	$4.001\times 10^{3}\pm 1.899\times 10^{2}$	0.525±0.025	278.209±13.770	1.187±0.060
11	$6.142\times 10^{-7}\pm 2.975\times 10^{-8}$	2.989±0.149	12.294±0.602	1.175±0.054	$2.945\times 10^{3}\pm 1.462\times 10^{2}$	0.526±0.026	208.552±10.454	1.121±0.058
12	$6.655\times 10^{-7}\pm 3.264\times 10^{-8}$	2.986±0.153	12.255±0.634	1.171±0.053	$3.008\times 10^{3}\pm 1.557\times 10^{2}$	0.526±0.027	209.127±10.545	1.123±0.057
13	$5.967\times 10^{-7}\pm 2.993\times 10^{-8}$	2.995±0.147	12.296±0.617	1.171±0.055	$3.068\times 10^{3}\pm 1.532\times 10^{2}$	0.527±0.026	223.678±10.903	1.138±0.057
14	$6.366\times 10^{-7}\pm 3.207\times 10^{-8}$	2.979±0.144	12.300±0.598	1.169±0.054	$2.977\times 10^{3}\pm 1.450\times 10^{2}$	0.527±0.027	209.614±9.993	1.117±0.056
15	$5.948\times 10^{-7}\pm 3.064\times 10^{-8}$	2.987±0.149	12.315±0.615	1.168±0.055	$2.922\times 10^{3}\pm 1.413\times 10^{2}$	0.527±0.026	222.062±10.873	1.154±0.057
16	$6.338\times 10^{-7}\pm 3.098\times 10^{-8}$	2.977±0.143	12.303±0.627	1.173±0.052	$3.208\times 10^{3}\pm 1.620\times 10^{2}$	0.524±0.026	224.222±10.977	1.135±0.057
17	$6.049\times 10^{-7}\pm 2.971\times 10^{-8}$	2.997±0.147	12.274±0.588	1.173±0.053	$3.547\times 10^{3}\pm 1.751\times 10^{2}$	0.525±0.026	244.535±11.554	1.156±0.055
18	$6.458\times 10^{-7}\pm 3.237\times 10^{-8}$	2.975±0.145	12.314±0.620	1.173±0.055	$2.985\times 10^{3}\pm 1.552\times 10^{2}$	0.522±0.025	209.307±10.592	1.123±0.054
19	$5.852\times 10^{-7}\pm 2.874\times 10^{-8}$	2.982±0.143	12.312±0.593	1.172±0.054	$3.192\times 10^{3}\pm 1.585\times 10^{2}$	0.524±0.025	224.393±11.033	1.094±0.054
20	$6.028\times 10^{-7}\pm 2.983\times 10^{-8}$	2.994±0.144	12.295±0.614	1.167±0.055	$3.532\times 10^{3}\pm 1.763\times 10^{2}$	0.527±0.025	248.378±12.065	1.154±0.056

**Table A6:** The same procedures as in Table A3 were applied to the *C. moschata* species.

	*q*-Gamma				Inverse *q*-Gamma			
**CHR**	$A_{G}$	$a_{G}$	$\sigma_{G}$	$q_{G}$	$A_{\mathrm{IG}}$	$\alpha_{\mathrm{IG}}$	$\sigma_{\mathrm{IG}}$	$q_{\mathrm{IG}}$
1	$6.508\times 10^{-7}\pm 3.261\times 10^{-8}$	2.981±0.146	12.318±0.614	1.170±0.055	$3.014\times 10^{3}\pm 1.528\times 10^{2}$	0.525±0.026	208.699±10.713	1.117±0.055
2	$5.945\times 10^{-7}\pm 3.004\times 10^{-8}$	2.983±0.150	12.255±0.627	1.171±0.055	$2.810\times 10^{3}\pm 1.427\times 10^{2}$	0.524±0.025	209.805±10.839	1.126±0.055
3	$6.775\times 10^{-7}\pm 3.310\times 10^{-8}$	2.988±0.147	12.267±0.611	1.170±0.052	$3.367\times 10^{3}\pm 1.706\times 10^{2}$	0.524±0.027	242.470±12.013	1.159±0.057
4	$6.425\times 10^{-7}\pm 3.298\times 10^{-8}$	2.992±0.141	12.256±0.638	1.170±0.054	$2.946\times 10^{3}\pm 1.399\times 10^{2}$	0.526±0.026	209.261±10.465	1.124±0.054
5	$6.306\times 10^{-7}\pm 3.181\times 10^{-8}$	2.976±0.147	12.276±0.614	1.170±0.055	$3.332\times 10^{3}\pm 1.636\times 10^{2}$	0.523±0.026	225.113±11.021	1.127±0.055
6	$6.020\times 10^{-7}\pm 2.931\times 10^{-8}$	2.992±0.148	12.325±0.631	1.174±0.054	$3.316\times 10^{3}\pm 1.638\times 10^{2}$	0.526±0.026	241.424±11.991	1.160±0.057
7	$6.456\times 10^{-7}\pm 3.202\times 10^{-8}$	2.988±0.146	12.295±0.622	1.168±0.052	$3.138\times 10^{3}\pm 1.619\times 10^{2}$	0.528±0.025	208.060±10.257	1.116±0.054
8	$6.267\times 10^{-7}\pm 3.003\times 10^{-8}$	2.985±0.143	12.266±0.629	1.171±0.054	$2.940\times 10^{3}\pm 1.505\times 10^{2}$	0.526±0.026	209.010±10.454	1.128±0.055
9	$5.722\times 10^{-7}\pm 2.943\times 10^{-8}$	3.001±0.146	12.290±0.591	1.169±0.052	$3.329\times 10^{3}\pm 1.640\times 10^{2}$	0.526±0.025	253.320±12.660	1.177±0.059
10	$6.285\times 10^{-7}\pm 3.179\times 10^{-8}$	2.985±0.151	12.301±0.610	1.168±0.054	$3.160\times 10^{3}\pm 1.631\times 10^{2}$	0.524±0.027	209.186±10.450	1.101±0.054
11	$5.913\times 10^{-7}\pm 2.892\times 10^{-8}$	2.979±0.145	12.240±0.614	1.168±0.054	$3.119\times 10^{3}\pm 1.519\times 10^{2}$	0.523±0.025	228.948±11.847	1.140±0.057
12	$6.453\times 10^{-7}\pm 3.262\times 10^{-8}$	2.982±0.149	12.301±0.620	1.171±0.054	$3.123\times 10^{3}\pm 1.541\times 10^{2}$	0.525±0.026	211.091±10.676	1.110±0.054
13	$5.884\times 10^{-7}\pm 2.832\times 10^{-8}$	2.970±0.151	12.267±0.642	1.170±0.054	$3.555\times 10^{3}\pm 1.805\times 10^{2}$	0.524±0.026	245.276±12.358	1.144±0.059
14	$6.144\times 10^{-7}\pm 3.129\times 10^{-8}$	2.978±0.146	12.245±0.603	1.168±0.055	$2.919\times 10^{3}\pm 1.412\times 10^{2}$	0.524±0.025	208.038±10.411	1.122±0.056
15	$5.996\times 10^{-7}\pm 3.118\times 10^{-8}$	2.982±0.153	12.313±0.611	1.168±0.054	$2.749\times 10^{3}\pm 1.424\times 10^{2}$	0.524±0.026	209.581±10.296	1.142±0.057
16	$6.098\times 10^{-7}\pm 3.227\times 10^{-8}$	2.995±0.147	12.299±0.617	1.172±0.056	$3.433\times 10^{3}\pm 1.748\times 10^{2}$	0.524±0.026	233.568±12.013	1.130±0.055
17	$6.325\times 10^{-7}\pm 2.996\times 10^{-8}$	2.980±0.142	12.284±0.615	1.171±0.053	$3.297\times 10^{3}\pm 1.663\times 10^{2}$	0.525±0.025	221.457±10.878	1.118±0.056
18	$6.389\times 10^{-7}\pm 3.120\times 10^{-8}$	2.981±0.144	12.308±0.617	1.171±0.055	$3.013\times 10^{3}\pm 1.516\times 10^{2}$	0.523±0.026	208.723±10.208	1.114±0.054
19	$5.940\times 10^{-7}\pm 3.001\times 10^{-8}$	2.984±0.150	12.278±0.609	1.174±0.052	$3.070\times 10^{3}\pm 1.541\times 10^{2}$	0.526±0.025	222.439±10.900	1.134±0.056
20	$1.455\times 10^{-6}\pm 7.255\times 10^{-8}$	2.686±0.134	12.301±0.606	1.207±0.059	$4.365\times 10^{3}\pm 2.241\times 10^{2}$	0.528±0.024	296.115±15.331	1.189±0.057

**Table A7:** The same procedures as in Table A3 were applied to the *Cucurbita pepo* species.

	*q*-Gamma				Inverse *q*-Gamma			
**CHR**	$A_{G}$	$a_{G}$	$\sigma_{G}$	$q_{G}$	$A_{\mathrm{IG}}$	$\alpha_{\mathrm{IG}}$	$\sigma_{\mathrm{IG}}$	$q_{\mathrm{IG}}$
1	$6.171\times 10^{-7}\pm 3.091\times 10^{-8}$	2.980±0.141	12.262±0.617	1.175±0.055	$3.063\times 10^{3}\pm 1.541\times 10^{2}$	0.525±0.025	209.370±10.632	1.120±0.057
2	$5.976\times 10^{-7}\pm 3.004\times 10^{-8}$	2.974±0.139	12.269±0.597	1.170±0.055	$2.990\times 10^{3}\pm 1.479\times 10^{2}$	0.522±0.026	208.945±10.598	1.112±0.056
3	$6.206\times 10^{-7}\pm 2.988\times 10^{-8}$	2.983±0.137	12.255±0.603	1.173±0.053	$3.016\times 10^{3}\pm 1.519\times 10^{2}$	0.527±0.025	208.831±10.467	1.130±0.055
4	$5.776\times 10^{-7}\pm 2.841\times 10^{-8}$	2.986±0.144	12.286±0.624	1.170±0.054	$2.981\times 10^{3}\pm 1.487\times 10^{2}$	0.526±0.025	209.237±10.066	1.115±0.055
5	$6.014\times 10^{-7}\pm 3.191\times 10^{-8}$	2.981±0.148	12.288±0.609	1.170±0.055	$2.950\times 10^{3}\pm 1.428\times 10^{2}$	0.524±0.025	209.034±10.637	1.118±0.052
6	$6.753\times 10^{-7}\pm 3.312\times 10^{-8}$	2.988±0.148	12.278±0.605	1.166±0.055	$3.504\times 10^{3}\pm 1.745\times 10^{2}$	0.528±0.025	209.254±10.435	1.113±0.058
7	$6.694\times 10^{-7}\pm 3.220\times 10^{-8}$	2.974±0.150	12.302±0.616	1.168±0.054	$3.289\times 10^{3}\pm 1.591\times 10^{2}$	0.524±0.024	208.556±10.929	1.107±0.057
8	$5.717\times 10^{-7}\pm 2.892\times 10^{-8}$	2.975±0.149	12.285±0.622	1.175±0.055	$2.611\times 10^{3}\pm 1.348\times 10^{2}$	0.524±0.025	209.373±9.912	1.138±0.056
9	$7.129\times 10^{-7}\pm 3.538\times 10^{-8}$	2.984±0.146	12.271±0.643	1.171±0.054	$3.458\times 10^{3}\pm 1.749\times 10^{2}$	0.526±0.026	209.003±10.615	1.116±0.057
10	$5.951\times 10^{-7}\pm 3.026\times 10^{-8}$	2.985±0.146	12.345±0.614	1.169±0.055	$2.968\times 10^{3}\pm 1.460\times 10^{2}$	0.524±0.026	209.462±10.334	1.116±0.055
11	$1.464\times 10^{-6}\pm 7.125\times 10^{-8}$	2.767±0.136	12.319±0.613	1.173±0.053	$3.445\times 10^{3}\pm 1.794\times 10^{2}$	0.526±0.026	231.581±11.564	1.091±0.054
12	$6.232\times 10^{-7}\pm 3.308\times 10^{-8}$	2.997±0.150	12.308±0.613	1.168±0.053	$3.050\times 10^{3}\pm 1.458\times 10^{2}$	0.527±0.026	208.665±10.225	1.113±0.056
13	$6.060\times 10^{-7}\pm 3.198\times 10^{-8}$	2.979±0.150	12.268±0.596	1.178±0.057	$2.928\times 10^{3}\pm 1.463\times 10^{2}$	0.522±0.025	209.873±10.115	1.126±0.055
14	$6.588\times 10^{-7}\pm 3.406\times 10^{-8}$	2.966±0.145	12.338±0.631	1.171±0.054	$3.264\times 10^{3}\pm 1.620\times 10^{2}$	0.527±0.026	210.521±10.453	1.107±0.055
15	$6.099\times 10^{-7}\pm 2.902\times 10^{-8}$	2.984±0.142	12.290±0.613	1.175±0.055	$3.004\times 10^{3}\pm 1.460\times 10^{2}$	0.525±0.026	209.403±10.791	1.119±0.056
16	$6.112\times 10^{-7}\pm 2.964\times 10^{-8}$	2.988±0.143	12.312±0.585	1.167±0.053	$2.997\times 10^{3}\pm 1.481\times 10^{2}$	0.525±0.025	208.459±10.559	1.120±0.057
17	$5.964\times 10^{-7}\pm 2.999\times 10^{-8}$	2.989±0.151	12.296±0.640	1.169±0.054	$2.922\times 10^{3}\pm 1.498\times 10^{2}$	0.524±0.025	208.371±10.415	1.116±0.055
18	$6.194\times 10^{-7}\pm 3.193\times 10^{-8}$	2.967±0.147	12.332±0.620	1.167±0.056	$3.226\times 10^{3}\pm 1.574\times 10^{2}$	0.522±0.027	211.364±10.848	1.123±0.056
19	$5.717\times 10^{-7}\pm 2.802\times 10^{-8}$	2.979±0.153	12.323±0.628	1.174±0.055	$2.596\times 10^{3}\pm 1.303\times 10^{2}$	0.526±0.025	210.284±10.298	1.139±0.056
20	$5.720\times 10^{-7}\pm 2.901\times 10^{-8}$	2.980±0.147	12.340±0.599	1.172±0.056	$3.645\times 10^{3}\pm 1.794\times 10^{2}$	0.525±0.026	273.876±13.334	1.177±0.060

The Bayesian factors and evidence values for each chromosome of the species *C. maxima*, *C. moschata*, and *C. pepo* are presented in Table A8. The Bayesian evidence for the q−gamma distribution is shown in column $\ln(\epsilon_{1})$; the Bayesian evidence for the inverse q−gamma distribution is shown in column $\ln(\epsilon_{2})$; and the Bayesian factor is shown in column $\ln(B_{1,2})$. The Jeffreys’ scale found in Table 1 of the main text can be used to analyze the data shown in the table. As we can see, the findings make it impossible to identify any of the suggested distributions as superior. Consequently, either can be used to study the exon length distributions of the species analyzed here without bias.

**Table A8:** The table presents the Bayesian evidence and the Bayes factors for each chromosome of the *C. maxima*, *C. moschata*, and *C. pepo* species. The column $\ln(\epsilon_{1})$ presents the Bayesian evidence for the *q*-Gamma distribution, $\ln(\epsilon_{2})$ presents the Bayesian evidence for the inverse *q*-Gamma distribution, and $\ln(B_{1,2})$ presents the Bayes factor.

	*C. maxima*			*C. moschata*			*C. pepo*		
CHR	$\ln\left(\epsilon_{1}\right)$	$\ln\left(\epsilon_{2}\right)$	$\ln\left(B_{1,2}\right)$	$\ln\left(\epsilon_{1}\right)$	$\ln\left(\epsilon_{2}\right)$	$\ln\left(B_{1,2}\right)$	$\ln\left(\epsilon_{1}\right)$	$\ln\left(\epsilon_{2}\right)$	$\ln\left(B_{1,2}\right)$
1	−0.065±0.016	−0.020±0.010	−0.044±0.006	−0.095±0.024	−0.020±0.010	−0.074±0.014	−0.101±0.037	−0.020±0.010	−0.081±0.027
2	−0.076±0.027	−0.020±0.010	−0.055±0.017	−0.090±0.022	−0.019±0.009	−0.070±0.012	−0.079±0.020	−0.020±0.010	−0.058±0.010
3	−0.086±0.022	−0.020±0.010	−0.065±0.012	−0.083±0.020	−0.020±0.010	−0.062±0.010	−0.086±0.025	−0.021±0.010	−0.065±0.015
4	−0.091±0.029	−0.020±0.010	−0.071±0.019	−0.091±0.019	−0.020±0.010	−0.071±0.009	−0.077±0.023	−0.020±0.010	−0.057±0.013
5	−0.085±0.025	−0.021±0.010	−0.064±0.015	−0.074±0.017	−0.020±0.010	−0.054±0.007	−0.087±0.025	−0.019±0.010	−0.068±0.015
6	−0.090±0.027	−0.020±0.009	−0.069±0.017	−0.119±0.028	−0.020±0.010	−0.099±0.018	−0.095±0.026	−0.023±0.010	−0.072±0.016
7	−0.092±0.031	−0.020±0.010	−0.072±0.021	−0.089±0.020	−0.021±0.010	−0.068±0.010	−0.089±0.030	−0.022±0.010	−0.067±0.020
8	−0.064±0.019	−0.021±0.010	−0.043±0.009	−0.085±0.024	−0.020±0.010	−0.065±0.014	−0.077±0.022	−0.018±0.009	−0.058±0.012
9	−0.074±0.026	−0.020±0.010	−0.053±0.016	−0.084±0.024	−0.019±0.010	−0.065±0.014	−0.094±0.016	−0.023±0.010	−0.070±0.006
10	−0.074±0.013	−0.021±0.009	−0.053±0.003	−0.084±0.019	−0.021±0.010	−0.063±0.009	−0.082±0.016	−0.020±0.010	−0.062±0.006
11	−0.093±0.041	−0.020±0.010	−0.073±0.031	−0.094±0.023	−0.020±0.010	−0.074±0.013	−0.108±0.024	−0.046±0.010	−0.062±0.014
12	−0.115±0.023	−0.021±0.010	−0.094±0.013	−0.086±0.022	−0.020±0.009	−0.065±0.012	−0.087±0.025	−0.020±0.010	−0.067±0.015
13	−0.088±0.026	−0.019±0.010	−0.069±0.016	−0.076±0.025	−0.020±0.010	−0.055±0.015	−0.093±0.022	−0.020±0.010	−0.073±0.012
14	−0.104±0.016	−0.020±0.010	−0.084±0.007	−0.099±0.025	−0.020±0.010	−0.078±0.015	−0.090±0.017	−0.021±0.010	−0.069±0.007
15	−0.072±0.021	−0.019±0.010	−0.052±0.011	−0.081±0.020	−0.020±0.010	−0.061±0.010	−0.089±0.026	−0.020±0.010	−0.068±0.016
16	−0.086±0.020	−0.020±0.010	−0.065±0.010	−0.104±0.026	−0.020±0.010	−0.084±0.016	−0.082±0.028	−0.020±0.010	−0.062±0.018
17	−0.083±0.022	−0.020±0.010	−0.063±0.012	−0.097±0.033	−0.020±0.010	−0.076±0.023	−0.070±0.021	−0.019±0.010	−0.050±0.011
18	−0.093±0.021	−0.020±0.010	−0.072±0.011	−0.095±0.025	−0.020±0.010	−0.075±0.015	−0.083±0.021	−0.022±0.010	−0.061±0.011
19	−0.061±0.032	−0.010±0.010	−0.051±0.022	−0.076±0.025	−0.019±0.010	−0.056±0.015	−0.077±0.022	−0.018±0.009	−0.058±0.012
20	−0.086±0.025	−0.020±0.010	−0.066±0.015	−0.099±0.024	−0.020±0.010	−0.078±0.014	−0.083±0.018	−0.019±0.010	−0.064±0.008

## Appendix B. Deduction of the Inverse *q*-Gamma and *q*-Gamma Distributions

### Appendix B.1. Inverse Gamma Superstatistics

Based on the results obtained from the time series analysis, we will establish the size distribution from the temporal evolution of exon sizes. We start with the assumption that the local distribution within a specific time interval *T* follows from a conditional distribution for *l* given a value of ξ, namely:

(A1) $$f\left(l|\xi \right)=\frac{1}{\Gamma (k)}{\left(\frac{k}{\xi }\right)}^{k}x^{k-1}\exp\left(-\frac{k}{\xi }l\right).$$

Next, we introduce the stationary inverse gamma distribution to incorporate fluctuations in the local mean ξ of the exon sizes:

(A2) $$p\left(\xi \right)=\frac{\omega^{\mu }}{\Gamma (\mu )}\xi^{-\mu -1}\exp\left(-\omega /\xi \right).$$

Consequently, the joint probability of obtaining a specific value of *l* and ξ, denoted as P(l,ξ), is given by:

(A3) P(l,ξ)=f(l|ξ)p(ξ).

Moreover, the marginal probability of obtaining a given value of *l*, independent of ξ, is expressed as:

(A4) $$p\left(l\right)=\int_{0}^{\infty }f\left(l|\xi \right)p\left(\xi \right)d\xi .$$

By applying Equations (A1) and (A2) to Equation (A4) and performing the integration, we obtain the following expression:

(A5) $$p\left(l\right)=\frac{\Gamma (k+\mu )}{\Gamma (k)\Gamma (\mu )}\left(\frac{k}{\omega }\right){\left(\frac{k}{\omega }l\right)}^{k-1}{\left(1+\frac{k}{\omega }l\right)}^{-k-\mu }.$$

Now, it is appropriate to introduce some changes of variables as follows:

(A6) $$\begin{array}{c} \frac{k}{\omega }=\frac{q-1}{\sigma },\;k=\frac{1}{q-1}-\mu ,\;a=k-1. \end{array}$$

Therefore, Equation (A5) can be expressed as $p_{G}\left(l\right)$:

(A7) $$p_{G}\left(l\right)=A_{G}{\left(\frac{l}{\sigma }\right)}^{a}{\left[1+\left(1-q\right)\left(\frac{l}{\sigma }\right)\right]}^{\frac{1}{1-q}}.$$

Equation (A7) can be expressed in terms of the *q*-exponential as follows:

(A8) $$p_{G}\left(l\right)=A_{G}{\left(\frac{l}{\sigma }\right)}^{a}\exp_{q}\left(-\frac{l}{\sigma }\right).$$

Here, the *q*-exponential is defined as:

(A9) $$\exp_{q}\left(-\frac{l}{\sigma }\right)={\left[1+\left(1-q\right)\left(\frac{l}{\sigma }\right)\right]}^{\frac{1}{1-q}}.$$

where $p_{G}\left(l\right)$ represents the *q*-Gamma probability density, and $A_{G}$ is defined as:

(A10) $$A_{G}=\frac{{\left(q-1\right)}^{a+1}\Gamma \left(\frac{1}{q-1}\right)}{\sigma \Gamma \left(\frac{1}{q-1}-a-1\right)\Gamma \left(a+1\right)}.$$

### Appendix B.2. Gamma Superstatistics

We can create the discrete-time “temporal” evolution for the exon size distribution by applying the process described in Appendix B.1. Consequently, assuming a local distribution, a data window of size *T* is defined by the distribution of *l* for a given ξ

(A11) $$f\left(l|\xi \right)=\frac{\alpha^{\alpha +1}}{\xi \Gamma (\alpha +1)}{\left(\frac{l}{\xi }\right)}^{-\alpha -2}\exp\left(-\frac{\alpha \xi }{l}\right).$$

By following a rationale similar to the one described in Appendix B.1, to introduce the fluctuations in the local mean ξ of the exon sizes, we assume that ξ obeys the gamma distribution

(A12) $$p\left(\xi \right)=\frac{1}{\omega^{\delta }\Gamma \left(\delta \right)}\xi^{\delta -1}\exp\left(-\frac{\xi }{\omega }\right).$$

By using Equations (A11) and (A12) to the conditional probability equation represented by Equation (A4), as well as carrying out the integration, we obtain

(A13) $$p_{IG}\left(l\right)=\frac{\Gamma (\alpha +\delta +1)}{\omega^{\delta }\Gamma \left(\alpha +1\right)\Gamma \left(\delta \right)}{\left(\alpha \omega \right)}^{\alpha +1}x^{-\alpha -2}{\left(1+\frac{\alpha \omega }{l}\right)}^{-\alpha -\delta -1}.$$

Using the change of variables

(A14) $$\begin{array}{c} \alpha \omega =\sigma \left(q-1\right),\;\delta =\frac{1}{q-1}-\alpha -1 \end{array}$$

Equation (A13) can be rewritten in the form

(A15) $$p_{IG}\left(l\right)=A_{IG}{\left(\frac{l}{\sigma }\right)}^{-\alpha -2}{\left[1-\left(1-q\right)\left(\frac{\sigma }{l}\right)\right]}^{\frac{1}{1-q}}$$

Here, $P_{IG}\left(l\right)$ is the inverse *q*-Gamma probability density function. It is worth noting that Equation (A15) can be represented in terms of the *q*-exponential function in the form

(A16) $$p_{IG}\left(l\right)=A_{IG}{\left(\frac{l}{\sigma }\right)}^{-\alpha -2}\exp_{q}\left(-\frac{\sigma }{l}\right).$$

The *q*-exponential is defined as

(A17) $$\exp_{q}\left(-\frac{\sigma }{l}\right)={\left[1-\left(1-q\right)\left(\frac{\sigma }{l}\right)\right]}^{\frac{1}{1-q}}.$$

and $A_{IG}$ is given by

(A18) $$A_{IG}=\frac{\Gamma \left(\frac{1}{q-1}\right)}{\sigma \Gamma \left(\alpha +1\right)\Gamma \left(\frac{1}{q-1}-\alpha -1\right)}{\left(\frac{1}{q-1}\right)}^{-\alpha -1}.$$
